# Multifunctional in vitro, in silico and DFT analyses on antimicrobial BagremycinA biosynthesized by *Micromonospora chokoriensis* CR3 from *Hieracium canadense*

**DOI:** 10.1038/s41598-024-61490-9

**Published:** 2024-05-14

**Authors:** Rabia Tanvir, Saadia Ijaz, Imran Sajid, Shahida Hasnain

**Affiliations:** 1 Institute of Microbiology (IOM), University of Veterinary and Animal Sciences (UVAS), Lahore, 54000 Punjab Pakistan; 2 Department of Microbiology and Molecular Genetics, The Women University, Multan, 66000 Punjab Pakistan; 3 Institute of Microbiology and Molecular Genetics (IMMG), University of the Punjab, Quaid-e-Azam Campus, Lahore, 54590 Punjab Pakistan

**Keywords:** Drug discovery, Medicinal chemistry

## Abstract

Among the actinomycetes in the rare genera, *Micromonospora* is of great interest since it has been shown to produce novel therapeutic compounds. Particular emphasis is now on its isolation from plants since its population from soil has been extensively explored. The strain CR3 was isolated as an endophyte from the roots of *Hieracium canadense,* and it was identified as *Micromonospora chokoriensis* through 16S gene sequencing and phylogenetic analysis. The in-vitro analysis of its extract revealed it to be active against the clinical isolates of methicillin-resistant *Staphylococcus aureus* (MRSA) and *Candida tropicalis* (15 mm). No bioactivity was observed against Gram-negative bacteria, *Escherichia coli* ATCC 25922, and *Klebsiella pneumoniae* ATCC 706003. The *Micromonospora chokoriensis* CR3 extract was also analyzed through the HPLC-DAD-UV–VIS resident database, and it gave a maximum match factor of 997.334 with the specialized metabolite BagremycinA (BagA). The in-silico analysis indicated that BagA strongly interacted with the active site residues of the sterol 14-α demethylase and thymidylate kinase enzymes, with the lowest binding energies of − 9.7 and − 8.3 kcal/mol, respectively. Furthermore, the normal mode analysis indicated that the interaction between these proteins and BagA was stable. The DFT quantum chemical properties depicted BagA to be reasonably reactive with a HOMO-LUMO gap of (ΔE) of 4.390 eV. BagA also passed the drug-likeness test with a synthetic accessibility score of 2.06, whereas Protox-II classified it as a class V toxicity compound with high LD_50_ of 2644 mg/kg. The current study reports an endophytic actinomycete, *M. chokoriensis,* associated with *H. canadense* producing the bioactive metabolite BagA with promising antimicrobial activity, which can be further modified and developed into a safe antimicrobial drug.

## Introduction

Natural products such as secondary metabolites help their biosynthesizing organisms cope with biotic and abiotic stresses as well as act as biostimulants and biocontrol agents^1^. Apart from their role in various developmental stages and helping the producer strain cope in their habitat, these metabolites can be exploited for wide-ranging applications in therapeutic industries. Actinomycetes are prolific producers of secondary metabolites that have wide-ranging applications in the medical and pharmaceutical industries^2^. They are estimated to produce about 55% of the antibiotics in the world^3^ with potent therapeutic qualities and suitable pharmacokinetic characteristics needed for clinical use^2,4^. Among the genera, *Streptomyces* contributes 75%, and non*-Streptomyces* species contribute 25% of these antibiotics^3^. Therefore, the prospects of finding new compounds from them are of great interest. Being major members of the soil community, actinomycetes interact with other members, which increases the likelihood of finding compounds with new chemical structures. For this purpose, researchers have been putting intense efforts into finding rare actinomycetes, particularly those that are residing in novel niches. The search for actinomycetes has been conducted on various soils in a variety of geographical locations^5^. Many rare actinomycetes belonging to the genera *Amycolatopsis*, *Nocardia*, *Pseudonocardia,* and *Micromonospora* have been reported from such locations; therefore, attention has now been turned towards isolating them from plants^2^. The microorganisms in the inter or intracellular plant tissues are known as endophytes. They improve the fitness of the plant by creating a plethora of bioactive compounds^6^. The plant environment is considered an untapped niche because only 1% of the total population of microbes residing in it has been explored so far, and the majority of them (70%) are identified as fungi^7^. Bioprospecting for endophytic actinomycetes in plants is of particular interest since the plants may possess a unique internal environment^8^ that may support the production of compounds with novel chemical structures^6^.

The genus *Micromonospora* belongs to the family *Micromonosporaceae,* and it produces no aerial mycelium. Up until now, about 65 species have been reported^9^ from different environments such as soil, sediments, water, and root nodules^10^ that are reported to produce antibiotics such as diazepinomicin, lomaiviticins A and B, lupinacidins A and B, netamicin, and tetrocarcin A^9^. Most recently, antimicrobial metabolites such as sagamycin, gentamicin, halomicin, ivermectin, mutamicin, and mycinamicin have also been identified in them^11^. Since this genus is second to *Streptomyces* as a source of novel metabolites^12^, new species from it remain a focus of research^9^. Recent studies have reported various species of *Micromonospora* residing as endophytes in plants and have characterized their active metabolites. One such study by Nafei et al.^13^ reported *Micromonospora* sp. in the medicinal plants of Egypt to be producing antitumor compounds such as queuine, indole-3-carboxyaldehyde, brevianamide F, palitantin, diethyl phthalate, nandrolon, S-adenosylmethioninaminium, and heptelidic acid. Another study described two novel anthraquinones and antitumor compounds, lupinacidins A and B, from *Micromonospora lupini* found in the roots of the plant *Lupinus angustifolius* ^14^. The species *Micromonospora chalcea* was reported from the roots of a cucumber plant that were observed to be producing antifungal agents against the phytopathogen *Pythium aphanidermatum*^15^. A novel sp. in the *Micromonospora* genus, *Micromonospora terminaliae* sp. nov., was reported from the medicinal plant *Terminalia mucronata* that possessed the capability of producing antimicrobial peptides, lanthipeptides^16^. *Micromonospora chokoriensis* was first isolated as a novel isolate from the sandy soil in Chokoria, Bangladesh^17^. Later, it was reported from the mountain soil in Thailand^10^ and from the desert soils of Chile and China^11,12^. Previously a study^18^ reported the isolation of *M. chokoriensis* strain B020 as an endophyte from wild medicinal plants. Although no compounds were identified from it, however it was observed to be active against mastitogens.

Considering the low frequency of isolation of the *Micromonospora* genus from plants along with the possibility of it producing novel metabolites, our study evaluated the in vitro activity of *M. chokoriensis* CR3, a strain residing as an endophyte in *Hieracium canadense*. The study also investigated its active metabolites through HPLC-DAD-UV–VIS analysis using a non-target approach for detecting secondary metabolites directly from the culture extracts and identified a compound, BagA, that was observed to be active against Gram-positive bacterial and yeast pathogens. The in-silico analysis of BagA using molecular docking, DFT, and ADMET revealed its drug-like properties, which indicated its capability to be converted into a promising antimicrobial drug.

## Materials and methods

### Isolation of endophytes

Healthy plants of *H. canadense* were collected from the area around Girl’s Hostel No. 8 (31° 29 N, 74° 18 E) situated in the University of the Punjab, Quaid-e-Azam campus, Lahore, Pakistan. The plant was carefully uprooted, keeping the tissue intact, and placed in labeled bags. It was then washed thoroughly under tap water to remove any soil particles and adhered epiphytes. The plant parts were cut into 0.5 cm segments and surface sterilized using a five-step process as described^19^. Briefly, the segments were placed in 70% ethanol for 5 min and 0.9% sodium hypochlorite solution for 20 min. The disinfectants were removed by washing three times with autoclaved distilled water. Later, the tissues were immersed in 10% NaHCO_3_ for 10 min, then washed once using autoclaved distilled water. The cut parts were placed on three media: actinomycetes isolation agar (Difco laboratories), glycerol casein KNO_3_ agar^20^, and rice agar (Rice water, agar 18.0 g/L, pH 7.3–7.5)^19^. To restrict endophytic fungal growth, cycloheximide (50 µg/ml) was added to the media, and to limit epiphytic bacterial growth, tetracycline (20 µg/ml) was added. The plates were incubated at 28 ºC for 7–21 days and observed at regular intervals for rough, powdery, and embedded growth. A negative control with plant tissue segments rolled and plated on the three media plates was also prepared, and it was incubated at 28 °C to ensure optimum surface sterilization ^21^.

### Morphological and physiological characterization

*Micromonospora chokoriensis* CR3 was characterized through its morphological and physiological characteristics following its subculturing on glucose yeast extract malt extract agar (GYM)^22^. The morphological characteristics included growth patterns and colony characteristics such as shape, size, margin, texture, color of substrate, and aerial mycelium, as well as specific pigment production^9^. The physiological characteristics were observed according to the International Streptomyces Project (ISP), which included the formation of melanin^22^ and the utilization of carbohydrates and similar compounds, mainly sugars^23^. Additionally, nine physiological tests^22^ were also carried out.

### 16S rRNA gene sequencing

For sequencing, the DNA was extracted according to the method described by Tanvir et al.^19^. Briefly, approximately 0.5 g of mycelia was taken in an eppendorf from a well-grown GYM agar plate. The mycelium was washed with 400 µl of Tris EDTA (TE) buffer (Thermo Fisher Scientific, USA) and centrifuged at 10,000 rpm for 5 min. The supernatant was discarded and 400 µl of TE buffer was added again along with 20 µl of lysozyme (50 mg/ml) (Thermo Fisher Scientific, USA). It was incubated at 37 °C for 4 h. After the incubation, 50 µl of sodium dodecyl sulfate (SDS) (20%) and 5 µl of proteinase K (20 mg/ml) (Thermo Fisher Scientific, USA) were added, and the eppendorf was incubated at 37 °C for 4 h. About 400 µl of phenol, chloroform, and isopropanol (25:24:1) solution was added, and centrifugation was done at 12,000 rpm for 10 min. The resulting upper layer was collected in a separate eppendorf, and this step was repeated. Absolute ethanol (2 ×) was added, and it was left at − 20 °C overnight. The pellet was taken after centrifugation at 12,000 rpm for 15 min, and it was washed with 70% ethanol. The DNA was air dried, re-suspended in 50 µl TE buffer, and stored at -20 °C. It was then sent to a commercial sequencing service (Macrogen Sequencing Service, Seoul, South Korea) that carried out the PCR and 16S rRNA analysis to reveal the genus of the strain. The sequence was checked through BLASTN^24^, and the type strains of the genus that gave 99% homology were compared by building a phylogenetic tree using Mega 7^25^. A closely related genus, *Luedemannella helvata,* was used as an outgroup. The sequence was deposited in GenBank, and the accession number was obtained^26^.

### Small-scale cultivation and extract preparation

*Micromonospora chokoriensis* CR3 was sub cultured on GYM agar medium for 7–21 days at 28 °C. To confirm its purity, the subculture was observed microscopically. This subculture was used to inoculate 2 × 300 ml of GYM broth (pH 7.8) in 500 ml Erlenmeyer flasks by cutting out a block from the well-grown GYM agar plate from the 7–21-days incubation. The flasks were placed in an orbital shaking incubator at 180 rpm for 3–5 days at 28 °C. The resulting culture containing both broth and mycelium was subjected to ultra-sonication (J.P. Selecta S.A.) to breakdown the mycelium and release the resulting compounds in the broth. Then ethyl acetate (1:1) was added to it^27^, and the upper layer was collected. This layer was evaporated in a rotary evaporator (Heidolph, Germany), and the resulting extract was dissolved in absolute methanol. The extract was kept in a glass vial at 4 °C. The ethyl acetate extraction was done three times, and the yield of the extract was expressed as dry weight after calculation.

### Antimicrobial testing

For the antimicrobial activity, the agar-well diffusion method was applied as explained previously^28^, with slight modifications. The extract was checked against the clinical isolates *Pseudomonas* sp., *Enterobacter* sp., methicillin-resistant *Staphylococcus aureus* (MRSA), and *Candida tropicalis.* Other strains included *Staphylococcus aureus* ATCC 25923, *Escherichia coli* ATCC 25922, *Klebsiella pneumoniae* ATCC 706003, *Bacillus subtilis DSM 10* (ATCC 6051), *Escherichia coli* K12 (W1130), *Saccharomyces cerevisiae* ATCC 9080, and microalgae *Chlorella vulgaris.* About 60 µl of the crude extract (5 mg/mL) was loaded in the wells (5 mm). The plates were left at room temperature for 2 h for the extract to diffuse. They were then incubated at 30 °C for the yeast strain and at 37 °C for the bacterial strains for 18–24 h. The *C. vulgaris* test plate was incubated under a light intensity of 3000 lx for 96 h at room temperature. The resulting zones of inhibition were measured in mm(s). The minimal inhibitory concentration (MIC) of the CR3 extract was determined using the broth microdilution method as described^29^. The lowest concentration that completely inhibited the growth of the test organisms was taken as the MIC.

### Thin layer chromatography (TLC) bioautography

For the extract, bioautography was done as previously described^9^. Briefly, the test organism (*B. subtilis* DSM 10) was suspended in normal saline (10 ml, 0.85%) and compared with the 0.5 McFarland turbidity standard (BD Diagnostics, USA). The suspension (1 ml) was added to 14 ml of nutrient agar (0.6%) (Merck, Germany) (1:100). Thin layer chromatography (TLC) was carried out on a RP silica gel plate (Merck, Germany) using a MeOH/H_2_O (8:2) solvent system^9^ with the procedure as described by Kirchner^30^. The bands were marked under UV 254 and 365 nm, and the plate was cut into two parts. One part was used for staining using an anisaldehyde/H_2_SO_4_ solution, and the other plate was exposed to the UV for 15 min for sterilization. The nutrient agar seeded previously with *B. subtilis* DSM 10 was poured on the TLC plate. It was left to solidify and then incubated at 37 °C for 24 h. Following incubation, the plate was sprayed with a thiazolyl blue tetrazolium blue (MTT) (0.5 mg/mL) solution, and the color formation around the bands was observed. The yellow color indicated the bands with bioactivity, whereas the purple color indicated the bands with no activity.

### Preparative thin layer chromatography (pTLC)

The pTLC was carried out using 20 × 20 cm RP silica gel glass plates (Merck, Germany) as described by Sherma and Fried^31^, and the plates were developed using the solvent system MeOH/H_2_O in a ratio of 8:2. The bands were marked under the UV wavelengths 254 and 365 nm, and the target band was scrapped using a scalpel and crushed into a powder. It was added to absolute methanol and then filtered using a syringe filter (Agilent, USA) and kept in a glass vial at 4 °C.

### HPLC-DAD-UV–VIS analysis

HPLC-DAD-UV–VIS analysis of the extract was carried out using Agilent 1200 HPLC-DAD (Agilent, Waldbronn, Germany). The stationary phase was a 100 mm × 2 mm long 3 μm Nucleosil C18-column (Maisch, Ammerbuch, Germany) furnished with a 10 mm × 2 mm long pre-column with the same stationary phase. The mobile phase was a linear gradient elution from 10% eluent A (0.1% formic acid) to 100% eluent B (0.06% formic acid in acetonitrile) in 15 min. About 2.5 μl of the sample was injected into the column with an optimal flow rate of 400 µl/min. A diode array detector (UV/Vis DAD) was attached to the column, and the wavelengths of 230, 260, 280, 360, and 435 nm were monitored. For the peak acquisition, Agilent 6300 series ion trap software was used. The UV visible absorption spectra were analyzed by an automated search system that checked the purity of the peaks as well as ran a search in the in-house HPLC–UV-VIS database^32^. Further identification was done by matching their mass spectra with the mass spectra mentioned in the previous publication^33^.

### Molecular docking analysis

The antimicrobial and antifungal mechanisms of BagA were investigated by retrieving the 3D crystal structures of *Candida albicans* sterol 14-α demethylase (CaCYP51) (PDB ID: 5TZ1)^34^ and *Staphylococcus aureus* thymidylate kinase (SaTMK) (PDB ID: 4QGG)^35^ from the PDB database. For the comparison study, their human orthologs (hCYP51 and hTMK) were also retrieved with PDB IDs of 3JUV and 1E2Q, respectively. The structures of proteins were cleaned by eliminating co-crystallized ligands and water molecules except the heme of CYP51, which is the prosthetic group in the active site^36^. The active site coordinates were determined with Biovia Discovery Studio (21.1.0.20298). BagA (4-ethenylphenyl) 3-amino-4-hydroxybenzoate (compound CID: 10422480) was downloaded in 3D SDF format from the PubChem database, accessed on June 16, 2023. Its structure was then optimized with density functional theory (DFT) with a basis set of B3LYP/6-31G-d using Gaussian 09W software. Using AutoDock Tools (1.5.7), the proteins and ligand were prepared for docking simulations by adding polar hydrogens and Kollman charges to the proteins and Gasteiger charges to the ligand. A molecular docking was then carried out with AutoDock Vina (1.2.0) with grid centers x = 75, y = 65, and z = 5 and a grid box size of 22 Å × 22 Å × 22 Å for CaCYP51 and grid centers x = -77, y = 28, and z = -2 and a grid box size of 35 Å × 30 Å × 35 Å for hCYP51. Furthermore, grid center coordinates of x = 15, y = 0 and z = 3 and the grid box size of 21 Å × 21 Å × 21 Å were set for SaTMK and x = 14, y = 77 and z = 26 and the grid box size of 22 Å × 22 Å × 22 Å for hTMK. The top-ranked conformations with the lowest binding energies (ΔG) were selected and analyzed with Discovery Studio for bond distances and various ligand–protein interactions.

### MD simulation: normal mode analysis

To study the stability and motion of BagA-protein complexes, the lowest docking energies were evaluated by molecular dynamic simulation using the online iMODS server (https://imods.iqfr.csic.es/). The iMODS server works by performing molecular dynamic simulations through normal mode analysis by calculating the internal coordinates (torsional space)^37^. The stability of the protein-BagA complexes was analyzed with references to their chain deformability, B-factor, eigen values, covariance map, and elastic network model.

### DFT quantum chemical reactivity analysis

The chemical reactivity of BagA and its stability were determined by DFT by using B3LYP/6-31G-d as basis set ^38^, using Gaussian 09W software (Gaussian, Inc USA). The frontier molecular orbitals HOMO–LUMO, their energy gap and molecular electrostatic potential (MEP) were then visualized with GaussView 6.0.16 (64-bit Windows) software (Gaussian, Inc USA). Moreover, the following equations were used to calculate the chemical descriptors, for BagA.

$$HOMO\left( {Ionization\;Potential} \right) \ldots \; \ldots \; \ldots \; \ldots \; \ldots \; \ldots \; \ldots \; \ldots I = EHOMO$$

$$LUMO\left( {Electron\;Affinity} \right) \ldots \; \ldots \; \ldots \; \ldots \; \ldots \; \ldots \; \ldots \; \ldots A = - ELUMO$$

$$Energy\;gap \ldots \; \ldots \; \ldots \; \ldots \; \ldots \; \ldots \; \ldots \; \ldots \Delta E_{gap} = EHOMO - ELUMO$$

$$Chemical\;potential \ldots \; \ldots \; \ldots \; \ldots \; \ldots \; \ldots \; \ldots \; \ldots \mu = - \left( {1 + A} \right)/2$$

$$Global\;hardness \ldots \; \ldots \; \ldots \; \ldots \; \ldots \; \ldots \; \ldots \; \ldots \eta = \left( {1A} \right)/2$$

$$Global\;softness \ldots \; \ldots \; \ldots \; \ldots \; \ldots \; \ldots \; \ldots \; \ldots \sigma = 1/\eta$$

$$Electronegativity \ldots \; \ldots \; \ldots \; \ldots \; \ldots \; \ldots \; \ldots \; \ldots \chi = \left( {1 + A} \right)/2$$

$$Electrophilicity \ldots \; \ldots \; \ldots \; \ldots \; \ldots \; \ldots \; \ldots \; \ldots \omega = \mu^{2} /2\eta$$

### Pharmacokinetics and drug-likeness analysis

The drug-likeness and ADME properties of the DFT-optimized structure of BagA, utilizing its canonical SMILES, were evaluated using the SwissADME online web tool (http://www.swissadme.ch/). SwissADME computes multiple parameters of a potential drug, such as its physical and chemical attributes, its lipophilic characteristics, water solubility, pharmacokinetics, drug-likeness, and medicinal chemistry parameters^39^.

### Toxicity analysis

The in-silico toxicity analysis of BagA was first evaluated by the virtual lab ProTox-II (https://tox-new.charite.de/protox_II/), accessed on July 6, 2023, which predicts the toxicity parameters of small molecules through various models and endpoints^40^. Furthermore, the cardiotoxicity of BagA was also evaluated with Pred-hERG 5.0 (http://predherg.labmol.com.br/), which is an online web tool based on machine learning to identify possible hERG channel blockers and non-blockers promptly^41^. The in-silico testing of BagA was also done in various animal models for acute systemic and topical toxicity with the help of online web servers, BeeToxAI 1.0 (http://beetoxai.labmol.com.br/) and STopTox 6-Pack (https://stoptox.mml.unc.edu/). BeeToxAI is a QSAR tool based on machine learning developed to determine the toxicity of various compounds in honeybees^42^, whereas the STopTox tool is a battery of in-vivo assays also based on QSAR models derived from various experimental animal data in rats, rabbits, mice, and guinea pigs. These 6-pack assays are used by numerous regulatory organizations to assess various facets of acute toxicity in humans, such as toxicity upon inhalation, oral and dermal toxicity, eye irritability, and skin sensitization and irritation^43^.

### Statistical analysis

For each experiment, a triplicate study was carried out, and mean values were calculated along with their standard deviations (SD). They were evaluated further with the Least Significant Differences test through SPSS version 29.0 (SPSS, USA).

### Statement confirming experimental research and field studies on plants

We confirm that this study was in accordance with relevant institutional, national, and international guidelines and legislation of experimental research and field studies on plants (either cultivated or wild), including the collection of plant material.

### Statement of identification of plant

The plant used in this study was identified by Dr. Ghazala Nasim (Late) at the Institute of agriculture sciences, University of the Punjab, Lahore, Pakistan. A voucher specimen of the plant was not deposited in a publicly accessible herbarium because the herbarium in the University of the Punjab, Lahore, Pakistan is not accessible to the public and is for identifying plants.

## Results and discussion

### Isolation and characterization of *M. chokoriensis* CR3

Microbes such as actinomycetes produce natural products either to compete with other organisms or to use them at a particular developmental stage in their growth. Such specialized metabolites that arise from secondary metabolism are of great medicinal significance^2^. Among these actinomycetes are the rare group that are known to be difficult to cultivate, and the frequency of their isolation is lower than that of the *Streptomyces* strains. However, despite the difficulty in their isolation, they are the focus of study because of their ability to produce natural compounds with diverse structures and unusual bioactivities. The rare actinobacteria from underrepresented taxa, such as *Micromonospora*, are being screened in hitherto unexplored settings in the hopes of discovering novel therapeutic drugs^4^. Although rare actinomycetes are found in a variety of habitats, in recent years, it has become more and more difficult to find new natural products^44^. In order to uncover new microbial resources for potential bioactive agents, researchers have now turned towards the isolation of rare genera from plants^2^. Therefore, our study explored the rare actinomycetes belonging to the genus *Micromonospora,* particularly the species *M. chokoriensis*^45^. The strain was reported to have been isolated from the sandy soil near a waterfall in Chokoria, Bangladesh. It is interesting because our isolated strain, *M. chokoriensis* CR3, from the *H*. *canadensis* plant, was growing near a water stream that was used for irrigating the surrounding rice fields around the University of the Punjab, Lahore. Its roots, stem, and leaves were washed, surface sterilized, and plated on the three selective media. The *Micromonospora chokoriensis* CR3 strain was found to be growing on rice agar, and later it was sub-cultured on GYM agar. The strain exhibited the characteristic morphology of the genus *Micromonospora.* It was rough and leathery in appearance and lacked any visible sporulation. It displayed deep reddish-brown aerial mycelium and light reddish-brown substrate mycelium. Under the microscope, it was Gram-positive in long filamentous chains (Table. 1). In terms of morphological characteristics, the strain was previously reported^45^ to have a dark brown or cinnamon brown coloration on GYM agar^22^. Our strain, *Micromonospora chokoriensis* CR3, closely resembled the same color on similar media (Supplementary Figure S1a, b).

**Table 1:** Morphological and physiological characterization of *Micromonospora chokoriensis* CR3.

Morphological characteristics		
Size (mm)	10	
Shape	Circular	
Margin	Filamentous	
Texture	Rough	
Consistency	Hard/Embedded	
Sporulation	None	
Growth Pattern	Late growth/ Partitioned	
Substrate mycelium	Deep red to reddish brown	
Aerial mycelium	None	
Soluble Pigments	Yes	

Physiological characteristics		
Utilization of carbon sources	Growth on different days	Result
Glu	7d	+
	14d	+
Gal	7d	−
	14d	−
Ara	7d	−
	14d	−
Man	7d	−
	14d	−
Sor	7d	+
	14d	+
Suc	7d	–
	14d	−
Manni	7d	+
	14d	+
Raf	7d	+
	14d	+
Fru	7d	–
	14d	−
Lac	7d	+
	14d	+
Mel	8d,	–
	14d	–
UA	6d	+
	9d	+
	16d	+
UO	7d	–
	11d	–
	17d	–
FO	5d,	+
	9d	+
HEA	5d	+
	10d	+
HUA	5d	–
	9d	–
HT	2d	–
	5d	+

Tolerance to substances	Growth on different concentrations	Result
LV	0.1 ml	–
	0.5 ml	–
	1 ml	–
LY	0.1 ml	+
	0.5 ml	+
	1 ml	+
NaCl (4d)	0 g	+
	2 g	+
	3.5 g	+
	5 g	–
	6.5 g	–

Glu = Glucose; Gal = Galactose; Ara = Arabinose; Man = Mannose; Sor = Sorbitol; Suc = Sucrose.;

Manni = Mannitol; Raf = Raffinose; Fru = Fructose; Lac = Lactose; Mel = Melanin; UA = Utilization of Organic acids;

UO = Utilization of oxalate; FO = Formation of organic acids; HEA = Hydrolysis of esculin and arbutin; HUA = Hydrolysis of.

urea and allantoin; LV = lecithovitellin reaction; HT = Hemolysis test; LY = Resistance to lysozyme; NaCl = Resistance to.

NaCl (-) = negative results; ( +) = moderately positive; (+ +) = strongly positive; d = days.

**Figure 1:**
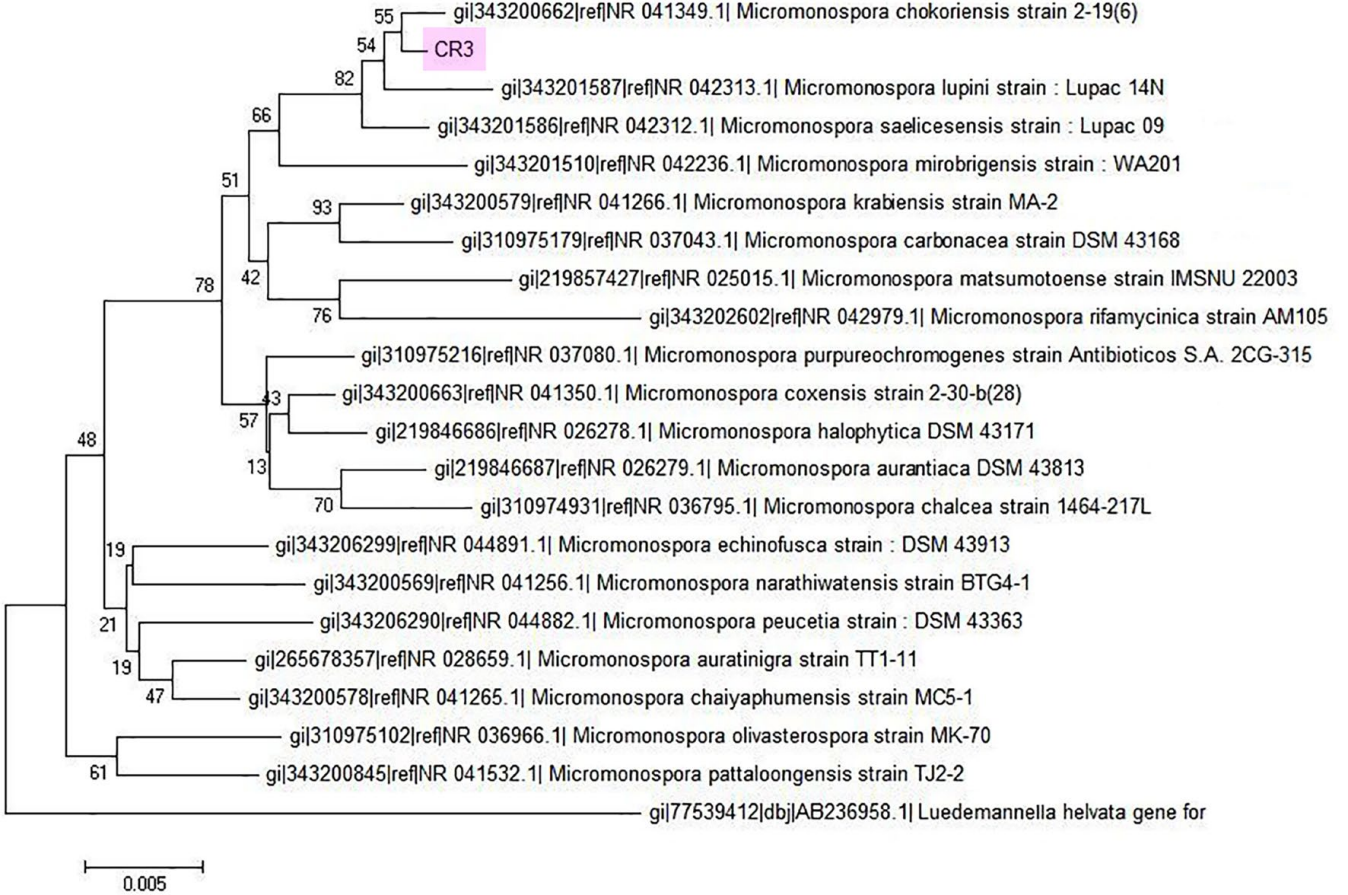
Neighbour Joining phylogenetic tree analysis of 16S rRNA gene sequence of *Micromonospora chokoriensis* CR3 and its twenty nearest homologues. *Micromonospora chokoriensis* CR3 clustered together with other *Micromonospora* sp. 16S rRNA gene sequences. 16S rRNA gene sequence of *Luedemannella helvata* was used as an out-group.

The strain was able to utilize glucose, sorbitol, mannitol, raffinose, and lactose as carbon sources. In the biochemical test, it tested negative for melanin production. Among the other tests, the strain *M. chokoriensis* CR3 displayed the ability to utilize organic acids, form organic acids, and hemolyze esculin and arbutin. Its physiological characteristics are described in detailed in Table 1. These characteristics were compared with those of the actinomycetes in the Bergey’s manual of determinative bacteriology^46^ and it confirmed that the strain did not belong to the genus *Streptomyces* but instead belonged to the rare actinomycetes genus such as *Micromonospora.* Further confirmation was done through 16S rRNA gene sequencing, and 1317 bp were obtained. In the BLASTN analysis, the sequence gave 99% homology to *Micromonospora* type strains. To narrow them down to a single species, a phylogenetic tree was constructed (Fig. 1). It indicated that the strain was related most closely to *Micromonospora chokoriensis* (GenBank accession no. KC191703).

### Antimicrobial activities of *M. chokoriensis* CR3

The antibacterial activity of *M. chokoriensis* CR3 extract was tested against three clinical isolates, five standard isolates, two yeasts, and one algal strain using the agar-well diffusion method and a broth microdilution assay. The *Micromonospora chokoriensis* CR3 extract displayed the most significant zone of inhibition (15.0 mm ± 0.577) against MRSA. For the remaining clinical isolates, the *M. chokoriensis* CR3 extract showed a zone of inhibition of 10 mm ± 1.00 against *Enterococcus* sp., but no inhibition was observed against *Pseudomonas* sp. In the case of the standard isolates, the *M. chokoriensis* CR3 extract showed a zone of inhibition of 12 mm ± 0.577 against *S*. *aureus* ATCC 25923 and 10 mm ± 1.00 against *B. subtilis* ATCC 6051. No zones were observed against *E. coli* ATCC 25922, *E. coli* K12, and *K. pneumonia* ATCC 706003. A zone of 15 mm ± 0.577 was observed against *S. cerevisiae* ATCC 9080 and 15 mm ± 0.577 against *C. tropicalis.* The *Micromonospora chokoriensis* CR3 extract displayed weak activity (10 mm ± 1.00) against *Ch. vulgaris.* It was observed that a small concentration (MIC value = 8 µg/ml) of the *M. chokoriensis* CR3 extract gave significant inhibitions against MRSA and *S*. *aureus* ATCC 25923. A significant MIC value, i.e., 8 µg/ml, was also observed against the yeast strain *C. tropicalis.* Moderate values (16 µg/ml) were observed against *Enterococcus* sp., *B. subtilis* ATCC 6051, *S. cerevisiae* ATCC 9080, and *Ch. vulgaris.* No MIC values were seen against *Pseudomonas* sp., *E. coli* ATCC 25922, *E. coli* K12, and *K. pneumonia* ATCC 706003.

Interestingly, the actinomycetes strain *Streptomyces* sp. Tü 4128, capable of producing the antimicrobial compound BagA, was observed to be only active against Gram-positive isolates and against fungi^33^. Similar results are indicated in our study, where *M. chokoriensis* CR3 was active against the Gram-positive pathogens MRSA and *S. aureus* as well as against the yeast pathogen *C. tropicalis*. Other studies reporting the isolation of *M. chokoriensis* reported no activity against Gram-positive pathogens, such as the study by Zhao et al.^47^, which reported the isolation of *M. chokoriensis as* an endophyte from the medicinal plant *Stellera chamaejasme* L., but the strain showed no bioactivity against *S*. *aureus* ATCC 25923 and *E. coli* ATCC 35218. Similarly, in another study by Zhao et al.^48^, no activity of *M. chokoriensis* was observed against *S*. *aureus* ATCC 25923 and *E. coli* ATCC 35218.

The possibility that the plant environment may play a role in the production of BagA cannot be ruled out. Our previous study on endophytic actinomycetes from the Asteraceae plant family showed the production of bioactive amide derivatives such as 7-Octadecenamide, 9-Octadecandienamide, and 12-Octadecandienamide^9^. Previous studies have shown that the amide part of the molecule confers antimicrobial activity^49^. Considering this, the *M. chokoriensis* strain in this study was also isolated from a plant belonging to the Asteraceae family that produces BagA, which is a phenol ester placed in the category of compounds that are linear amides. Therefore, it can be hypothesized that the internal environment of the plant may potentially play a role in the production of compounds in this category.

### Screening by TLC and bioautography

The TLC screening of the *M. chokoriensis* CR3 extract exposed the presence of medium polar and polar bands. On a reverse phase (RP) silica plate, the *M. chokoriensis* CR3 extract showed medium polar bands, whereas a polar band was also observed. The bands showed absorbance at UV 254 nm and relatively more absorbance at 365 nm (Supplementary Figure S2a, b). After staining with anisaldehyde/H_2_SO_4_ reagent, red and yellow colors were observed for the medium polar bands, and a purple color was observed for the polar band (Supplementary Figure S2c). In the bioautography, results for the *M. chokoriensis* CR3 extract showed that the medium polar and polar bands were bioactive against *B. subtilis* (Supplementary Figure S2d).

### HPLC-DAD-UV analysis

**Figure 2:**
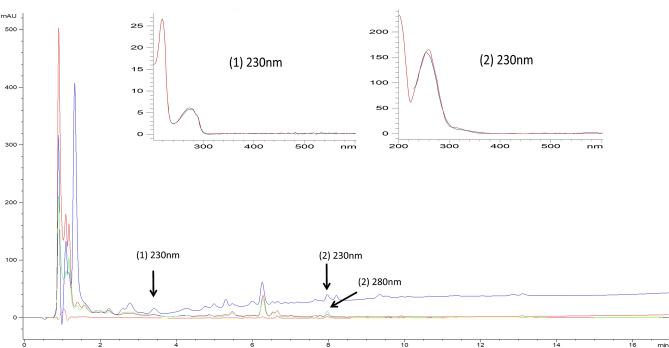
Total UV absorption spectra of *Micromonospora chokoriensis* CR3 at 230 (blue), 260 (red), 280 (light green), 360 (pink), 435 nm (green) wavelengths and an overlay of UV/Vis spectra match with NL 19KF (1) at 230 nm, retention time 3.59 min (blue = extract, red = database) and BagA (2) at 230, 280 nm, retention time 7.9 min (blue = extract, red = database).

HPLC-DAD analysis of the polar and medium polar bioactive bands was carried out using the screening strategy described by Bertasso et al.^33^. The strategy saves time and effort by employing a non-target approach for detecting secondary metabolites directly from the culture extracts of the isolated actinomycetes. The detection is done using reverse-phase HPLC connected to a diode array detector that screens a database with more than 600 reference compounds that are mostly antibiotics. Known compounds are detected by comparing their visible UV (UV–Vis) spectra using a match factor and their retention times. The match factors near or above 995 indicated maximum similarities with the reference compounds in the database. Using this strategy, one peak with a low match factor of 995.139 and retention times of 3.59 min was identified as NL 19 KF. Another peak with a high match factor of 997.334 at 7.9 min was identified as BagA (Fig. 2). The compound NL 19 KF was confirmed by the similarity in the retention times as previously described^50^. In the case of BagA, in addition to the retention time (7.9 min), the peak also gave an absorbance at 280 nm similar to that reported previously^33^. This further confirmed the presence of this compound in the extract. Other peaks that gave non-significant match factors included a peak that gave a 994.476 match factor with cephalosporin C^51^. Another peak gave a match factor of 990.794 and 990.021 for the compounds abyssomicin E and abyssomicin D; however, the retention times varied from those previously reported by Fiedler^52^ respectively.

The result from the HPLC analysis confirmed our results about the broad spectrum of activity of *M. chokoriensis* CR3. Previous studies have reported the antibiotic family of bagremycins (A-G) to be antimicrobial and antifungal^2^. Among them, BagA and BagB have been reported from *Streptomyces* sp. Tü 4128, and they were observed to be active against Gram-positive bacteria and fungi^53,54^. The study^33^ first reported its isolation from *Streptomyces* sp. Tü 4128, which displayed antimicrobial activity against *B. subtilis* DSM 10 (19 mm), *S. cerevisiae* ATCC 9080 (9 mm), and *C. albicans* Tü 164 (8 mm). Particularly, BagA was reported to exhibit activity against *C. albicans*^53^. It is interesting because genetically, *C. tropicalis* has the highest similarity to *C. albicans*^55^ as indicated in our study, where activity was indicative of *C. tropicalis*. Another study^56^ reported their production from *Streptomyces* sp. Q22 that was isolated as an endophyte from a mangrove plant. It is interesting that this study also mentioned the strong cytotoxic potential of bagremycins against glioma cell lines, with an IC_50_ value of 2.2 to 6.4 µM. This can be correlated to our previous study on the cytotoxic activity of the extract of *M. chokoriensis* CR3, which displayed strong cytotoxicity with an LC_50_ of 10 µg/ml^57^.

### Molecular docking analysis

For the molecular docking experiment, the compound BagA was selected based on its maximum similarity in the UV (UV–Vis) spectral database as well as similarity in its UV absorbance and retention time with previous studies^33^. The antibacterial and antifungal mechanisms of BagA were studied using two enzymes: Sterol 14-α demethylase (CYP51) of *Candida albicans* and Thymidylate Kinase (TMK) of *Staphylococcus aureus* MRSA252. CYP51 is one of the members of the heme-containing cytochrome-P450 oxidoreductase enzymes found in the outer membranes of the endoplasmic reticulum of the cells of fungi and other eukaryotes, including humans^58^. It works by catalyzing the methylation of lanosterol 14α in order to biosynthesize ergosterol, which is involved in the structural integrity and permeability of the cell membrane, without which the fungal cells do not survive^59^. Therefore, it can be a potential candidate for both systematic and topical clinical antifungal drugs^60^.

Thymidylate kinase (TMK) is a widely distributed enzyme that plays a key role in the biosynthesis of bacterial DNA, which in turn promotes cell growth and survival^61^. In the pyrimidine salvage pathway, this highly conserved enzyme catalyzes the synthesis of thymidine 5′-diphosphate (dTDP), converting it into thymidine 5’-triphosphate (dTTP)^62^. Thymidylate is an essential component of DNA that can only be biosynthesized with the help of dTTP^63^. When TMK activity is inhibited, the amount of thymidylate inside the cell decreases, which prevents DNA replication and results in cell death^62^. Thus, these CYP51 and TMK proteins can be viewed as crucial therapeutic targets for the synthesis and progression of novel antifungal and antibacterial drugs.

### CYP51-BagA complex

**Figure 3:**
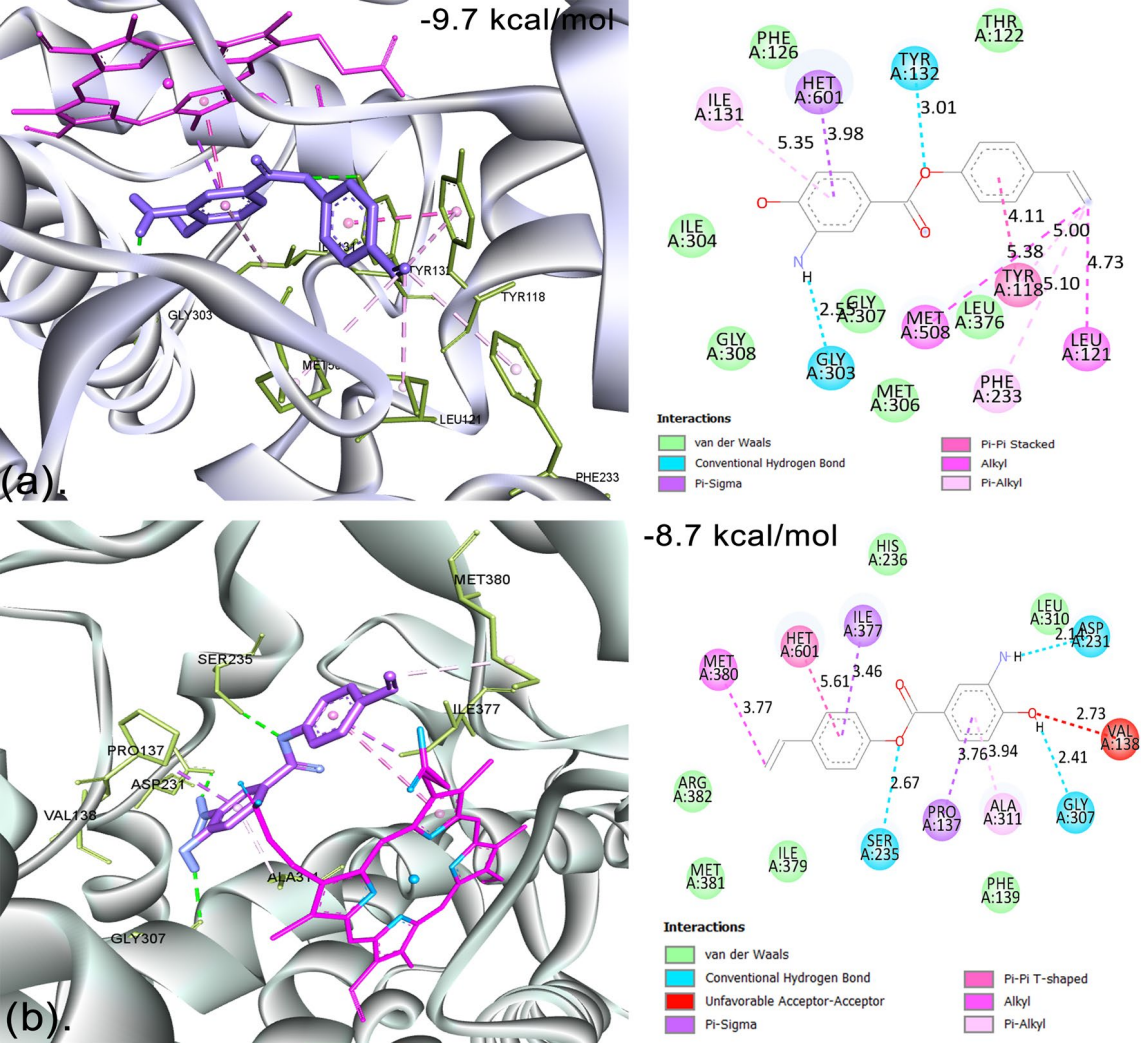
Molecular Docking Analysis of CaCYP51 and hCYP51 with BagA (**a**) The 3D and 2D molecular docking interactions of CaCYP51-BagA complex. (**b**) The 3D and 2D molecular docking interactions of hCYP51-BagA complex.

In the case of CaCYP51, the ligand BagA showed a docking score with the lowest binding energy of − 9.7 kcal/mol, as shown in Fig. 3a. It made two conventional hydrogen bonds through H27 of the enamine group with GLY:303 (LIG1:H (H donor)-GLY303:O (H acceptor)) with a bond distance of 2.55 Å and through O1 of the carboxyl group with TYR:132 (LIG1:O (H acceptor)-TYR132:O (H donor)) with a bond distance of 3.01 Å. A strong hydrophobic π-sigma bond was formed between the ND pyrrole ring of heme HET601:CMD and the π-orbital of BagA with a bond distance of 3.98 Å. Two hydrophobic π-alkyl bonds were formed between LIG1: π-orbital and ILE:131 and C19 of BagA and PHE:233 with bond distances of 5.35 and 5.10 Å, respectively. There were also two alkyl bond formations between C19 of BagA and MET:508 and LEU:121, with bond distances of 5.38 and 4.73 Å, respectively. TYR:118 of CaCYP51 made two interactions with BagA, one through a hydrophobic π–π-stacked interaction and the other with π-alkyl bonding with C19. Furthermore, various van der Waals forces were also seen interacting with the catalytic site residues involving THR:122, PHE:126, ILE:304, GLY:308, GLY:307, MET:306, and LEU:376. These results indicate that the inhibitory action of BagA against CaCYP51 is primarily due to hydrophobic interactions with the enzyme. Shi et al.^64^ also showed that the main mechanism by which inhibitors bind to CaCYP51 is through hydrophobic interaction. Blanco et al.^65^ also showed that CYP51 bonded with posaconazole through multiple hydrophobic interactions and one hydrogen bond. In a recent study, similar residues were seen interacting with various synthesized benzo[g]quinazolines (A-F) compounds^66^.

BagA showed the lowest binding energy of − 8.7 kcal/mol with hCYP51, where it interacted through different residues than CaCYP51, which consisted of three conventional hydrogen bonds: through H27 of the enamine group with ASP:231 with a bond distance of 2.14 Å, through H30 of the enol group with GLY:307 with a bond distance of 2.41 Å, and through O1 of the carboxyl group with SER:235, as shown in Fig. 3b. A hydrophobic π–π-T-shaped bond was formed between the pi-orbital of BagA and heme:601 with a bond distance of 5.61 Å. Two π-sigma bonds were formed between PRO:137 and the aromatic ring and between ILE:377 and the pi-orbital. A hydrophobic π-alkyl bond was also formed between ALA:311 and the aromatic ring of BagA (3.94 Å), whereas a single alkyl bond was formed between C19 and MET:380 with a bond distance of 3.77 Å. Various van der Waals forces were also seen interacting with the catalytic site residues involving HIS:236, ARG:382, MET:381, ILE:379, PHE:139, and LEU:310.

The CYP51 proteins are found in soluble form in prokaryotes, whereas they are membrane-bound in eukaryotes^67^. Cholesterol in mammals, ergosterol in fungus, and phytosterols in plants are the end products of sterol biosynthesis and are crucial structural elements of eukaryotic membranes^68^. Selectivity against microbial or fungal CYP51 is critical for treating human infections since co-inhibiting hCYP51 would prevent cholesterol synthesis and cause an uncontrollably high level of harmful sterols to build up^69^. In a previous study by Ogris et al.^70^, a derivative of pyridylethanol (phenylethyl) amine compound-24 was investigated for the selective inhibition of CaCYP51 from hCYP51. They showed that the halogenated ring extending into the pyrrole ring is the most important factor leading to the selective inhibition of CaCYP51. In the current study, the aromatic ring of BagA formed a strong hydrophobic interaction with heme, having a small bond distance of 3.98 Å as compared with hCYP51, where the bond distance was 5.61 Å, and thus could be further studied to see if this has any role in increasing the selectivity of BagA against CaCYP51.

### TMK-BagA complex

**Figure 4:**
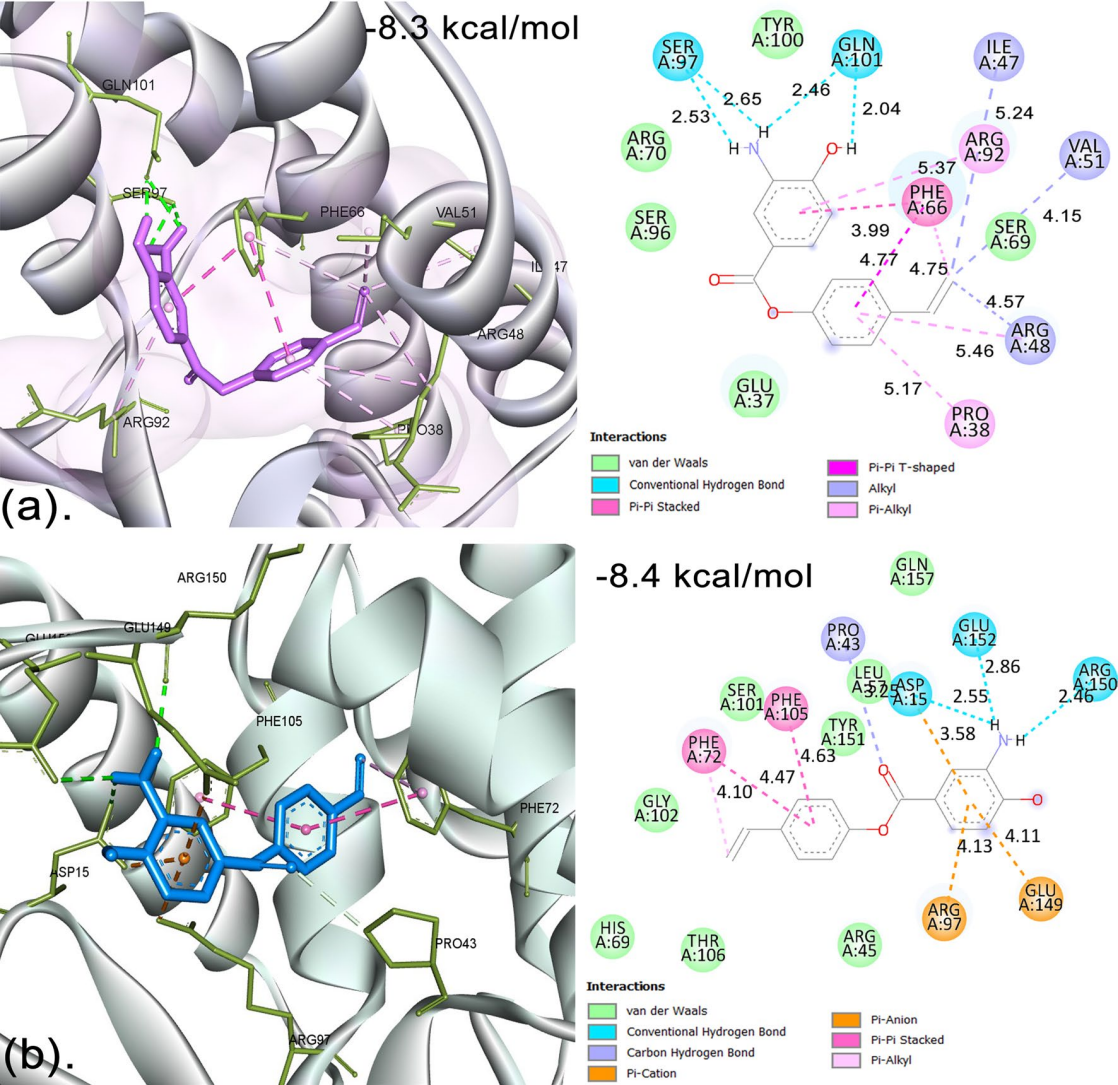
Molecular Docking Analysis of SaTMK and hTMK with BagA (**a**) The 3D and 2D molecular docking interactions of SaTMK-BagA complex. (**b**) The 3D and 2D molecular docking interactions of hTMK-BagA complex.

Upon docking, BagA showed a docking score with the lowest binding energy of -8.3 kcal/mol with SaTMK, as shown in Fig. 4a. SaTMK-BagA interactions consisted of two conventional hydrogen bonds through H27 and H28 of the enamine group with SER:97 (LIG1:H H-donor and SER97:OG H-acceptor), giving bond distances of 2.53 and 2.65 Å, respectively, and through H28 and 22 with GLN:101 (LIG1:H H-donor and GLN101:OE1 H-acceptor), with bond distances of 2.46 and 2.04 Å, respectively. Furthermore, various hydrophobic interactions like π–π-stacked, π–π-T-shaped, and π-alkyl bonds were formed between aromatic rings and C19 with PHE:66 (PHE:66-LIG1-orbitals and PHE:66-LIG1-C19), giving bond distances of 3.99, 4.77, and 4.75 Å, respectively. One π-alkyl bond with a distance of 5.37 Å was also formed with ARG92 (LIG1-ARG:92 π-orbitals). Two π-Alkyl bonds were further formed with ARG:48 and PRO:38 (LIG1-ARG48 and LIG1-PRO38 Pi orbitals) with distances of 5.46 and 5.17 Å, respectively. Moreover, C19 formed three alkyl bonds with ILE:47, VAL:51, and ARG:48, with bond distances of 5.24, 4.15, and 4.57 Å, respectively. BagA also formed van der Waals forces with TYR:100, ARG:70, SER:96, GLU:37, and SER:69. Similar results were also reported by Benselama et al.^71^, in which they showed that the inhibition of TMK was mainly due to non-covalent, hydrogen, and hydrophobic interactions. Moreover, Kawatkar et al.^72^ also showed that TMK interacts with hydrophobic PHE:66 and ARG:92, which are the crucial residues of this protein.

In the case of hTMK, BagA showed the lowest binding energy of − 8.4 kcal/mol, quite similar to SaTMK; however, BagA interacted with hTMK through different residues than SaTMK, which consisted of three conventional hydrogen bonds through H27 of the enamine group with ASP:15 and GLU:152, giving bond distances of 2.55 and 2.86 Å, respectively, and through H28 with ARG:150, giving a bond distance of 2.46 Å, as shown in Fig. 4b. One carbon–hydrogen bond was formed with PRO:43 at a distance of 3.25 Å, whereas two π–anion and one π–cation bond were formed between GLU:149, ASP:15, and ARG:97, respectively. Two π–π stacked bonds and one π-alkyl bond were formed between PHE:105, PHE:72, and PHE:72, respectively.

The TMK enzyme is widely distributed and can be found in prokaryotes, eukaryotes, and some viruses^73^. The bacterial (bTMK) and hTMKs are structurally and functionally similar; however, there are a few critical differences between them, such as the presence of 29 residues in the N-terminal region and the insertion of 12 and 8 residues at positions 117 and 146, respectively, in the hTMKs^74^. Furthermore, bTMKs crystallize only in the closed conformation both in the presence and absence of ligands, whereas the hTMKs structurally transform when they interact with the ligands through the loop comprising residues 181–197, including the main catalytic nucleophilic cysteine 195, which rotates 180° out of the active site in its unliganded state (inactive form)^74^. There are three highly conserved, distinct domains in the TMK that classify TMKs into type I and type II, which include: (i) the P-loop with consensus sequence Gxxx1xGKx; (ii) the DRX (X = Tyr, Phe) motif, in which the Mg^2+^ ions interact with Asp (D) residue, making the donor ATP and acceptor TMP come in close proximity; and (iii) the LID (ligand-induced degradation) domain, which is disordered and heterogeneous^75^. Type-I TMKs are present in eukaryotes and have an Arg residue that interacts with ATP at position × 1 in the consensus sequence of the P-loop, whereas class II TMKs from prokaryotes have a Gly residue in place of Arg at this position^62^. During phosphoryl transfer, the phosphate group is correctly positioned by the highly conserved Arg of the DRX motif, which also stabilizes the transition state^76^. One study showed that when Arg was mutated to Ala, it resulted in the complete loss of function of TMK^62^.

SaTMK shares a 19% sequence similarity with hTMPK and contains five basic residues in the LID region instead of the P-loop, thus suggesting the dTMP binding pocket to be most advantageous for the construction of selective inhibitors^77^. Furthermore, the highly conserved residue ARG:48 in SaTMK provides a key interaction in the Gram-positive binding pocket, which is not present in hTMK^78^. Similarly, in the current study, BagA also interacted through different residues in hTMK with no ARG:48 interactions.

In a previous study^72^, a structure-guided design approach was used to develop novel compounds 46 and 47, among others, with potent inhibitory activity against SaTMK, giving IC_50_ values of 1 nM each. These compounds also showed potent antibacterial activities against *S. aureus* ARC516 with MICs of 2 and 0.5 μg/mL, respectively, whereas compound 47 also showed potent activity against methicillin- and quinolone-resistant *S. aureus* ARC517 with a MIC of 0.5 μg/mL^72^. When these compounds were tested against hTMK, they exhibited a very high IC_50_ of 51,000 and 29,000 μg/mL, respectively, whereas no activity was detected against human A549 cell lines, indicating their selective activity against SaTMK^72^. Additionally, the X-ray crystal structures of these compounds bound with SaTMK (PDB ID: 4QGG and 4QGH) revealed interactions with major residues Phe66, Val51, and Arg48^62^, which were also seen interacting exclusively with SaTMK when BagA was used as a ligand as compared with hTMK and thus can be speculated to potentially target selectively against SaTMK with minimum toxicity against hTMK. Nevertheless, further work is still needed to assess the selective inhibition of SaTMK by BagA.

### MD simulation: normal mode analysis

**Figure 5:**
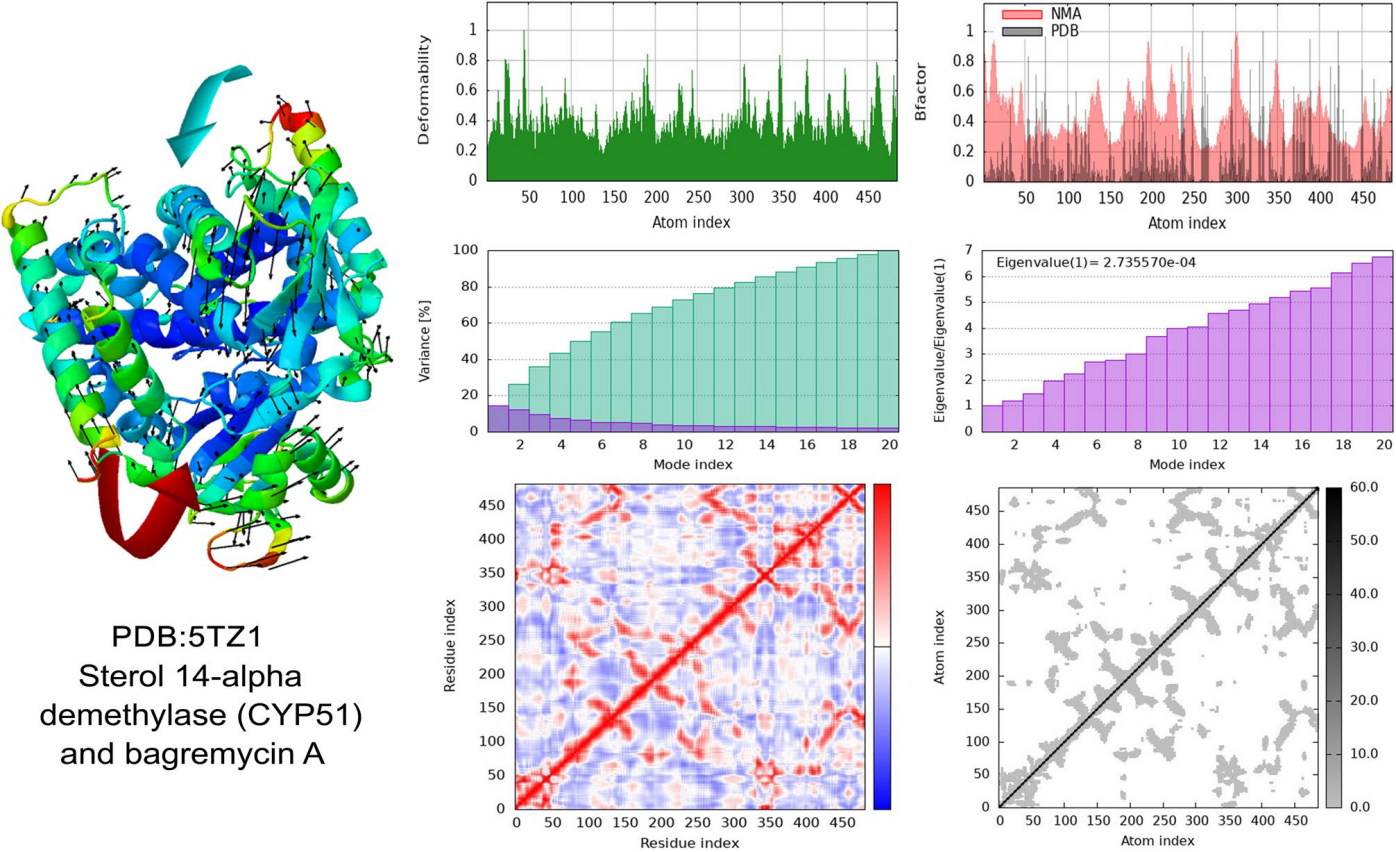
MD Simulation: normal mode analysis of CaCYP51-BagA complex. (**a**) Deformability (**b**) B-factor (**c**) Variance (**d**) Eigenvalues (**e**) Co-variance plot (**f**) Elastic network model.

Normal mode analysis (NMA) is an in-silico method by which protein conformational changes are studied^79^. The results obtained from MD simulation normal mode analysis of the CaCYP51-BagA complex gave a deformability index of 0.8 on average, as depicted in Fig. 5. The polypeptide chain deformability plot showed alterations in all the residues depicted by the tips of the peaks known as hinges where CaCYP51 and BagA interacted. Main-chain deformability refers to a molecule’s ability to shapeshift every amino acid residue in a polypeptide chain, a characteristic that explains how a molecule functions pertaining to its flexibility and stability^80^. The B-factor analyses showed reasonable atomic fluctuations, which indicated that the atoms behave in a highly stable and flexible manner. The variance graph showed higher levels of variance (approximately 18%), which made the eigenvalue low. The eigen value is indicative of the energy needed to distort the protein structure and serves as a reliable gauge of how stiff the protein residues are in motion^81^. For the current protein-BagA complex, the eigenvalue was calculated to be 2.7 × 10^−4^, which is considerably low, indicating that CaCYP51 can easily be deformed in the presence of BagA. In the MD simulation process, a compound with a low eigenvalue is considered to be more efficient, as less energy would be needed to deform a protein structure^82^. The covariance map, which shows the motion of positively correlating, non-correlating, and anti-correlating residues, is depicted by red, white, and blue colors, respectively^83^. The CaCYP51-BagA complex covariance map revealed distinct dark red areas with high degrees of positive correlation between residues, indicating that BagA and CaCYP51 formed stable interactions, which caused the residues to move in synchrony within a certain area. The concentration of blue area also showed significance as these interactions might restrict certain protein movements in other regions. The elastic network map of CaCYP51 indicated this protein to be less rigid, as depicted by light gray areas. To the best of our knowledge, no study has been done on the normal mode analysis of BagA with CaCYP51. However, the results can be compared with a study done on the antifungal polyprotein (RVT_1 region)-emetine complex, where it also showed a low eigenvalue (2.7 × 10^−5^) and high stability^84^.

**Figure 6:**
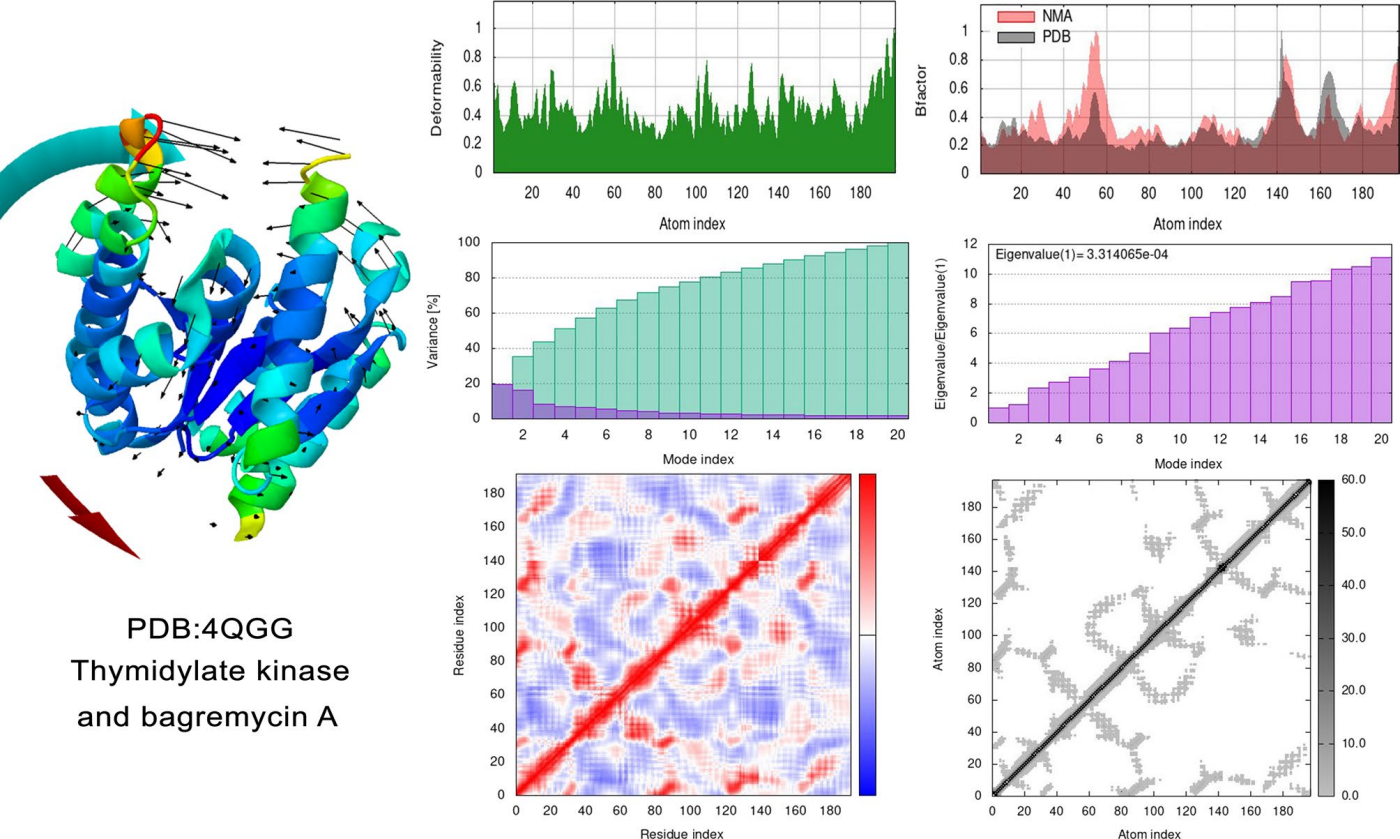
MD Simulation: normal mode analysis of SaTMK-BagA Complex. (**a**) Deformability (**b**). B-factor (**c**). Variance, (**d**). Eigenvalues (**e**). Co-variance plot (**f**). Elastic network model.

The NMA of the SaTMK-BagA complex showed similar results as CaCYP51-BagA. The polypeptide chain deformability plot of the docked SaTMK-BagA complex also showed alterations in all the residues, as shown in Fig. 6. The B-factor plot between the NMA and PDB simulations revealed a nearly identical pattern, indicating that the experimental data from the PDB and the simulation results of the test complex are comparable. The plot showed higher levels of variance (approximately 20%) with a low eigenvalue of 3.3 × 10^−4^, which indicated that the interactions between SaTMK and BagA were stable with simpler deformation. The covariance map of the SaTMK-BagA complex clearly showed dark red and light blue areas that contained a combination of more high- and low-interacting residues. Finally, the elastic network map of SaTMK indicated that this protein is very flexible, as depicted by its light gray coloring. To the best of our knowledge, no study has been done on the normal mode analysis of SaTMK; however, the results can be compared with the study done on antibacterial azo-thiazole derivatives against DNA gyrase by Aziz et al.^85^, where a low eigenvalue of 3.4 × 10^−3^ showed high stability.

### DFT quantum chemical analysis

**Figure 7:**
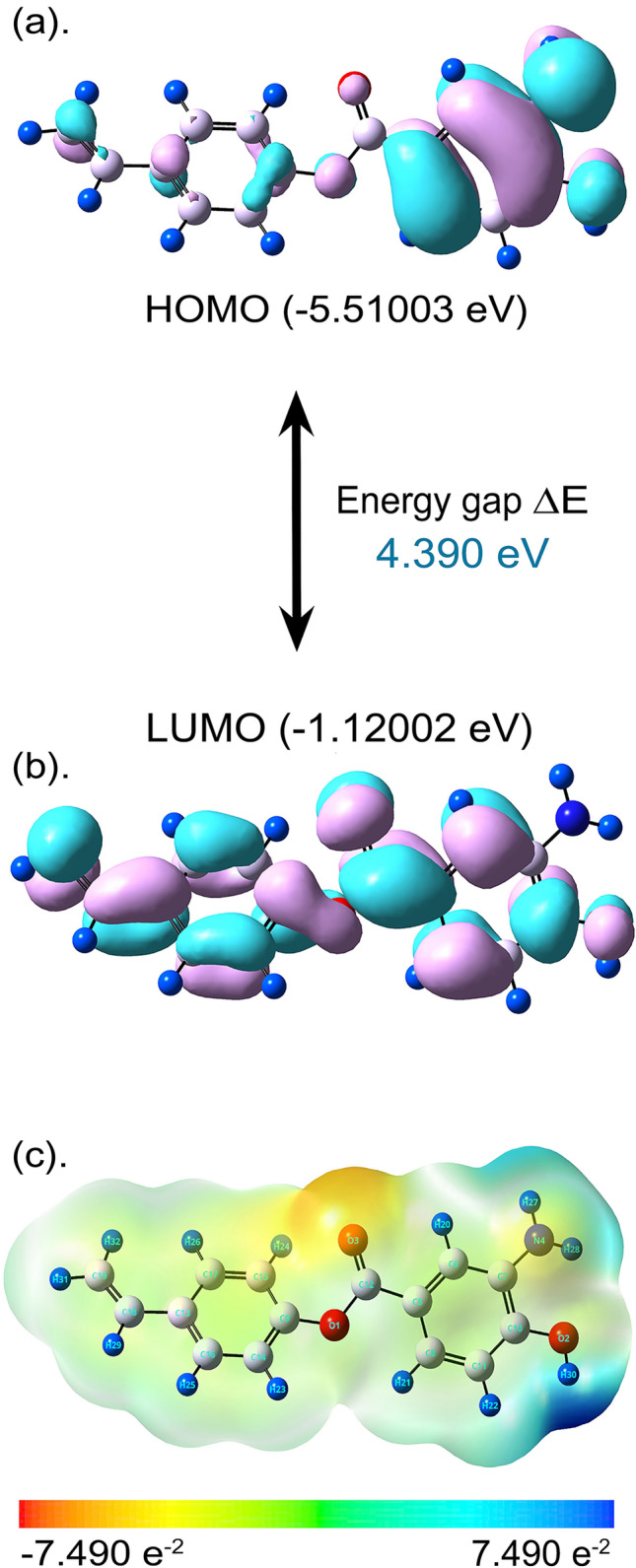
DFT Quantum Chemical Analysis of BagA (**a**) Frontier molecular orbital HOMO plot (**b**) Frontier molecular orbital LUMO plot. HOMO and LUMO plots are representing the positive (Pink) and negative (Cyan) phase distribution in molecular orbital wave function (**c**) Molecular electrostatic potential (MEP) plot showing the red (Electron rich) and blue (Electron poor) regions. The analysis was done with B3LYP/6-31G-d using Gaussian 09W software.

The DFT quantum mechanical analysis was performed to theoretically ascertain the electronic distribution of BagA by utilizing orbital energy calculations. The spatial distribution of the electron cloud on BagA can give an understanding of how it interacts with the proteins, giving insight into its binding mode. The frontier molecular orbitals (FMO), known as HOMO (the highest occupied molecular orbital) and LUMO (the lowest unoccupied molecular orbital), are the most important factors in defining the kinetic stability and the chemical reactivity of a molecule by showing how efficiently a molecule can transfer charge^86^. Thus, the most reactive regions in a molecule can be predicted using these FMOs. The HOMO and LUMO of BagA are shown in Fig. 7a and b. The HOMO was mostly found jumbled up on the right side of the molecules in the amino-hydroxybenzoate region, which showed this area to be electron dense, whereas the LUMO was seen spread across the whole molecule except the amino group. The HOMO–LUMO energy gap was also calculated, which is an important factor in defining the kinetic stability and intramolecular charge transfer potential of a molecule ^87^. A lower energy gap means that the electrons would easily be removed from HOMO (ground state) to LUMO (excited state)^88^. A molecule is classified as hard if it possesses large energy gaps because of difficulty in its polarization, as more energy is required for its excitation^89^. In the current study, BagA showed a HOMO–LUMO ground state energy gap (ΔE) of 4.390 eV, suggesting its high reactivity towards bonding with the proteins. Additionally, the descriptors of quantum chemical systems for BagA were also determined using HOMO–LUMO data, as given in Table 2. The electronegativity (χ), hardness (η), softness (σ), chemical potential (μ), and electrophilicity (ω) of BagA were calculated to be 3.315, 2.195, 0.510, -3.315, and 2.503 eV, respectively. The high and low values of μ and ω suggested good electrophilic and nucleophilic potentials of BagA, respectively. These results, along with a low HOMO–LUMO gap, show the molecule to be reasonably reactive.

**Table 2:** Quantum chemical parameters of BagA obtained from DFT analysis.

Sr. No	Parameter	Value
1	E_HOMO_ (I) (eV)	− 5.51003
2	E_LUMO_ (A) (eV)	− 1.12002
3	Energy Gap ΔE (eV)	4.390
4	Electronegativity χ (eV)	3.315
5	Global Hardness η (eV)	2.195
6	Global Softness σ (eV)	0.510
7	Chemical Potential μ (eV)	− 3.315
8	Electrophilicity ω (eV)	2.503
9	Dipole moment (Debye)	3.694
10	Electronic Energy (eV)	− 23,397.46

The molecular electrostatic potential (MEP) of BagA was also computed in order to study its chemical reactivity patterns. MEP is a 3D reactivity map that shows electronic density and is used to locate electrophilic and nucleophilic attack sites and hydrogen-bonding interactions in a molecule^90^. The MEP maps concurrently represent molecular shape, size, and areas representing positive, negative, and neutral electrostatic potential in the form of gradient colors that are very helpful when studying molecular interactions^91^. According to MEP, a molecule’s electrostatic potential rises starting from red, orange, yellow, green, and blue, whereby the maximum negative area is displayed in red, indicating a chemically favorable location for an electrophilic attack^92^. Conversely, the site that is conducive to a nucleophilic attack is indicated by the blue color, whereas zero or neutral potential zones are represented by the green color. In the present study, the B3LYP/6-31G-d method was applied for the construction of the MEP surface of BagA, which was mapped with color gradients ranging from − 7.490 × 10^−2^ to 7.490 × 10^−2^ as shown in Fig. 7c. The H30 of the enol group and the H27 and H28 of the amine group showed blue coloration, giving high positive potential and showing susceptibility to nucleophilic attack. Conversely, the red–orange region consisted of an oxygen atom attached to a carbonyl group (C12=O3) that showed moderate negative potential, showing susceptibility to the electrophilic attack. These results correlated with the results obtained from molecular docking analysis, where hydrogen bonding occurred from both of these regions. To the best of our knowledge, no study has been done on the DFT analysis or chemical reactivity of BagA; however, the results can be compared with the studies done on p-coumaric acid^93^ and benzoic acid derivatives^94^, as they are the precursors in the BagA biosynthetic pathway.

### Pharmacokinetics and drug-likeness analysis

The most crucial requirements for a potential drug candidate are to fulfill the ADME and drug-likeness properties. ADME is an abbreviation of four criteria: absorption, distribution, metabolism, and excretion, that are mainly investigated in pharmacokinetics. These are the primary steps in determining how a drug is metabolized in the body and its subsequent effects on the target organ and the body as a whole^95^. Drug-likeness, on the other hand, examines whether the drug candidate has characteristics that would make it an orally effective medicine based on Lipinski’s five rules^96^. The ADME and the drug-likeness predictions for the currently investigated compound BagA were done by a web-based online tool, SwissADME, that is given in Table 3. The results showed that the BagA compound had 2 H-bond donors and 3 H-bond acceptors. Its molecular weight was calculated to be 255.27 g/mol with 4 rotatable bonds, obeying the Veber and Muegge rules^97,98^. The molar refractivity of BagA was predicted to be 74.36, which obeyed the Ghose rule^99^. The TPSA was predicted to be 72.55 Å^2^ that obeyed the Veber rule^97^. Lipophilicity is another important physicochemical parameter that shows compound permeability across the cell membrane through passive diffusion^100^. In the case of BagA, the lipophilicity log *P*_o/w_ (logarithm of the n-octanol/water partition coefficient) was predicted to be 2.67, which was well in the range of 5, suggesting good permeability and absorption across the cell membranes. The water solubility Log *S* (ESOL -3.67, ALI -4.44, and SILICOS-IT -4.12) of BagA varied from soluble to moderately soluble. The blood–brain barrier (BBB) crossing and GI absorption of BagA were found to occur at high rates. This also shows that, apart from having antifungal properties, the inhibition of hCYP51 by BagA can also be potentially beneficial for treating various neurodegenerative diseases like Alzheimer’s by lowering cholesterol in the brain^101^.

**Table 3:** In-silico physicochemical and drug-likeness prediction of BagA by SwissADME.

Physicochemical characteristics									Drug-Likeness					Bioavailability Score (ABS)
Formula and Molecular weight	NRB^a^	NHD^b^	NHA^c^	TPSA^d^ (Å^2^)	Lipophilicity (Consensus Log *P*_*o/w*_)	Water Solubility			Lipinski	Ghose	Veber	Egan	Muegge	
						Log *S* (ESOL) Class	Log *S* (Ali) Class	Log *S* (SILICOS-IT)	Violations					
C_15_H_13_NO_3_ 255.27 g/mol	4	2	3	72.55	2.67	− 3.67 Soluble	− 4.44 Moderately Soluble	− 4.12 Moderately Soluble	Yes 0	Yes 0	Yes 0	Yes 0	Yes 0	0.55 Yes

ADME and Medicinal Chemistry														
Skin permeation Log *K*_p_ cm/s	BBB permeant	GI absorption	Substrate/ Inhibitor Interactions							Medicinal Chemistry				
			P-gp substrate	CYPIA2 inhibitor	CYP2C19 inhibitor	CYP2C9 inhibitor	CYP2D6 inhibitor	CYP3A4 inhibitor	PAINS	Brenk	Lead-likeness	Synthetic accessibility		
Yes − 5.56	Yes	Yes High	No	Yes	No	Yes	No	No	0 alerts	3 alerts: aniline, catechol, phenol-ester	Yes	2.06		

^a^NRB (Number of rotatable bonds), ^b^NHD (Number of H-bond donors), ^c^NHA (Number of H-bond acceptors), ^d^TPSA (Topological polar surface area.

Additionally, BagA was not predicted to be a P-glycoprotein (Pgp) substrate, so it is unlikely that Pgp overexpression would adversely influence its efficiency^102^. The skin permeability (Log *K*p) was predicted to be 5.56 cm/s, which showed the medium ability of BagA to penetrate skin as the permeability decreased with an increase in the negative value of Log *K*p. Furthermore, BagA was only found to be an inhibitor of CYP1A2 and CYP2C9. It obeyed the Lipinski rule of five, giving 0 violations and a bioavailability score of 0.55%, which means that it can be used as an oral therapeutic drug^103^. For medicinal chemistry parameters, no alert was observed for BagA in PAINS analyses that made it a genuine therapeutic drug candidate. Conversely, three alerts were observed in BRENK analyses due to aniline, catechol, and phenol ester moieties, which shows that the fragments of BagA could be putatively toxic; however, this may not be a serious concern as it depends on the nature of the structural moiety producing the alert^104^. Nonetheless, BagA passed the lead-likeness test with a synthetic accessibility score of 2.06. The current ADME results can be compared with the study of Kiani et al.^105^ for p-coumaric acid, as no study has been done on BagA for ADME analysis. Overall, the findings indicate that BagA is a highly promising candidate to be developed into a therapeutic drug.

### Toxicity analysis

Predicting the toxicity of new drugs is a fundamental step in their development. For the first time, the organ toxicities and toxicological endpoints of BagA and its LD_50_ were predicted in the current study using ProTox-II, which classifies drugs into various toxicity classes based on the LD_50_ (mg/kg body weight) values. The LD_50_ is defined as a lethal oral dose that can cause fatality in 50% of the test subjects upon exposure to a compound. The compounds are classified into six classes according to Globally Harmonized System (GHS) regulations, with Class 1 (LD_50_ ≤ 5) and Class II (5 < LD_50_ ≤ 50) being the most toxic, causing fatalities^106^. The Class III (50 < LD_50_ ≤ 300) indicates toxicity upon swallowing. Class IV (300 < LD_50_ ≤ 2000) indicates that it is harmful if swallowed. Class V (2000 < LD_50_ ≤ 5000) categorizes the compounds as having fewer chances of toxicity if swallowed, and Class VI (LD_50_ > 5000) classifies the compounds as non-toxic. In our study, BagA was classified as Class V by ProTox-II, giving an LD_50_ of 2644 mg/kg with an average similarity of 70%, and a prediction accuracy of 68%, as given in Table 4.

**Table 4:** In-silico toxicity prediction of BagA calculated with ProTox-II and Pred-hERG 5.0

Toxicity Analysis																					
Predicted Toxicity Class	Predicted LD_50_	Average similarity %	Prediction accuracy %	Toxicity End Points (Portability %)						Nuclear receptor signalling pathways (Probability %)							Stress response pathways (Probability %)				
				Cardiotoxicity (Pred-hERG)	Hepatotoxicity	Carcinogenicity	Immunotoxicity	Mutagenicity	Cytotoxicity	AhR^a^	AR^b^	AR-LBD^c^	Aromatase	ER^d^	ER-LBD^e^	PPAR-Gamma^f^	nrf2/ARE^g^	HSE^h^	MMP^i^	Tumor Suppressor p53	ATAD5^j^
5 Non-Toxic	2644 mg/kg	70	68	No (60)	Yes (62)	Yes (56)	Yes (52)	No (60)	No (69)	Yes (55)	No (88)	No (98)	No (80)	Yes (58)	No (61)	No (91)	No (92)	No (92)	Yes (55)	No (84)	No (51)

^a^Aryl hydrogen Receptor, ^b^Androgen Receptor, ^c^Androgen Receptor Ligand Binding Domain, Estrogen Receptor, ^e^Estrogen Receptor Ligand Binding Domain, ^f^Peroxisome Proliferator Activated Receptor Gamma.

According to the toxicity model report, BagA was causing hepatotoxicity, carcinogenicity, and immunotoxicity with a low prediction probability of 62, 56, and 52%, respectively. Similarly, Ouahabi et al.^107^ showed in a recent study that coumaric acid is a class V chemical with an LD_50_ value of 2850 mg/kg, which can induce carcinogenicity with a 68% probability. The toxicity of BagA was also detected against the aryl hydrocarbon receptor (AhR), estrogen receptor (ER), and mitochondrial membrane potential (MMP), with the same low prediction probability of 55, 58, and 55, respectively. Suppressed immunological responses, oxidative stress, and increased reactive oxygen species (ROS) formation are the main causes of drug-induced hepatotoxic effects^108^. As BagA is a phenol ester of 3-amino-4-hydroxybenzoic acid with a derivative of p-coumaric acid, its toxicity could be attributed to the pro-oxidant nature of the phenolic moiety, especially at higher concentrations, under alkaline conditions, or in the presence of heavy metal ions like Cu^2+^ or Fe^3+^^109^. Phenolic compounds are known to act as double-edged swords, acting both as antioxidants and pro-oxidants, which is mainly due to the number and positions of hydroxyl groups and to their redox metal (Cu, Fe) chelating capacity^110^.

BagA was also screened for potential cardiotoxicity by Pred-hERG, as the FDA mandates that every biomolecule undergo hERG safety testing prior to being employed as a drug candidate since blocking of the hERG protein channel has been linked to fatal cardiac arrhythmias^111^. The Pred-hERG classified BagA as non-cardiotoxic with a 60% prediction probability, as given in Table 5. As no study has been done on BagA Pred-HERG analysis, it was compared with Pred-HERG analysis of p-coumaric acid in a recent study done by Bhadana and Rani^112^, who also reported it to be a non-blocker. Interestingly, in a fairly recent study, for the first time, the cardioprotective properties of p-coumaric acid were studied by showing anti-tachycardial, anti-inflammatory, anti-ion pump dysfunction, and anti-electrolyte imbalance properties^113^.

**Table 5:** In-silico animal testing of BagA for acute systemic and topical toxicity.

Application	Assay type	Animal	Endpoint	Prediction	Contribution mapping	Confidence%
BeeToxAI 1.0	Acute Oral Toxicity Test (OECD 213)	Honey Bee (*Apis mellifera*)	Acute Oral Toxicity	Non-toxic _		65
	Acute Contact Toxicity Test (OECD 214)		Acute Contact Toxicity	Non-toxic _		70
STopTox (6-pack)	Acute inhalation toxicity test (OECD TG 403 and 436)	Rat	Acute Inhalation Toxicity	Non-Toxic _		78
	Acute oral toxicity test (OECD TG 401, 420, 423 and 425)	Rat	Acute Oral Toxicity	Non-Toxic **_**		70
	Dermal toxicity test (OECD TG 402)	Rabbit and Rat	Acute Dermal Toxicity	Non-Toxic _		65
	Draize test (OECD TG 405)	Rabbit	Eye Irritation and Corrosion	Toxic +		57
	LLNA test (OECD TG 429 and 442)	Mouse and Guinea Pig	Skin Sensitization	Sensitizer +		60%
	Draize test (OECD TG 404)	Rabbit	Skin Irritation and Corrosion	Non-Toxic _		80

For assessing the potential toxic effects of novel drug candidates, animal trials are necessary; however, the cost and use of animals are not always feasible^114^. In-silico procedures for toxicity testing present an alternative approach that enables the reduction of the associated time, cost, labor, and feasibility of animal experiments^115^. In the current study, BeeTox and STopTox (6-pack) online webtools were used to investigate the potential toxicity of BagA in various in-silico animal models. According to BeeTox, BagA was classified as non-toxic for both acute oral and contact toxicity with 65 and 70% prediction probabilities, as shown in Table 5. The toxicity was predicted by contour maps generated from QSAR models to visualize the atoms that were involved in toxicity. As shown in Table 5, the green areas in contour maps indicated a negative contribution for toxicity (i.e., pLD_50_ increases when the moieties are eliminated) and red indicated a positive contribution for toxicity (i.e., pLD_50_ decreases when the moieties are eliminated). Similarly, STopTox also classified BagA as non-toxic when acute inhalation, acute oral, acute dermal, and skin irritation/corrosion toxicity tests were performed. Conversely, BagA showed toxicity when eye irritation/corrosion and skin sensitization toxicity tests were performed with low prediction probabilities of 57 and 60%. Although this is the first work done on the *in-silico* animal testing of BagA, its toxicity profile can be compared with other phenolic compounds, such as cinnamate, which was also identified as a skin sensitizer in the original publication of StopTox^43^. Overall, the findings of the toxicity test suggested that BagA is mostly non-toxic; however, it may show serious organ toxicity at greater doses. Further research is still required to validate this and better understand the toxicity mechanism.

## Conclusion

In conclusion, in this study, a rare actinomycetes strain, *Micromonospora chokoriensis* CR3, demonstrated the ability to produce bioactive secondary metabolites. These metabolites displayed targeted inhibitory effects against Gram-positive bacteria such as MRSA and *S. aureus* (ATCC 25925), as well as the yeast pathogen *C. tropicalis,* but not the Gram-negative bacteria. The extract indicated a significant presence of the antibiotic BagA, which we perceive led to its narrow spectrum of antibacterial activity based on previous studies. The *in-silico* docking and simulation analysis of BagA further confirmed its stable interactions with the antibacterial (SaTMK 4QGG) and antifungal (CYP51 5TZ1) protein targets in *Staphylococcus aureus* subsp. *aureus* MRSA252 and *C. albicans* with low binding energies. DFT analysis showed it to be a reasonably reactive molecule. Pharmacokinetics and drug-likeness showed it to be a very promising drug-like molecule. Conversely, in the toxicity analysis, it showed some serious organ toxicity, but with a low prediction probability, which we speculate is due to its phenolic nature as BagA is a phenol ester. Thus, we conclude that *H. canadense* harbors a rare actinomycetes genus, *Micromonospora,* with the capability to produce potent antifungal and Gram-positive antibacterial metabolites, BagA, with promising potential to be converted into a safe and non-toxic antimicrobial drug.

### Supplementary Information

Supplementary Figures.

## Data Availability

The 16S rRNA gene sequence of the isolated endophyte *Micromonospora chokoriensis* from *Hieracium Canadensis* was deposited in the GenBank database under accession number KC191703 and its data is available at https://www.ncbi.nlm.nih.gov/nuccore/KC191703.
