# Supplementary material for: Multifunctional in vitro, in silico and DFT analyses on antimicrobial BagremycinA biosynthesized by Micromonospora chokoriensis CR3 from Hieracium canadense
Source: Sci Rep. 2024 May 14;14:10976. doi: 10.1038/s41598-024-61490-9 (PMC11093986; doi:10.1038/s41598-024-61490-9)
Supplement: Supplementary file 1 — Supplementary Figures. [file 41598_2024_61490_MOESM1_ESM.pdf]

SUPPLEMENTARY MATERIAL

Multifunctional *In Vitro*, *In Silico* and DFT Analyses on Antimicrobial BagremycinA Biosynthesized by *Micromonospora chokoriensis* CR3 from *Hieracium canadense*

[*]Rabia Tanvir[1], Saadia Ijaz[2], Imran Sajid[3] and Shahida Hasnain[3]

[1]*Institute of Microbiology (IOM), University of Veterinary and Animal Sciences (UVAS), 54000, Lahore, Punjab Pakistan.*

[2]*Department of Microbiology and Molecular Genetics, The Women University, 66000, Multan, Punjab, Pakistan*

[3]*Institute of Microbiology and Molecular Genetics (IMMG), University of the Punjab, Quaid-e-Azam Campus, 54590, Lahore, Punjab Pakistan.*

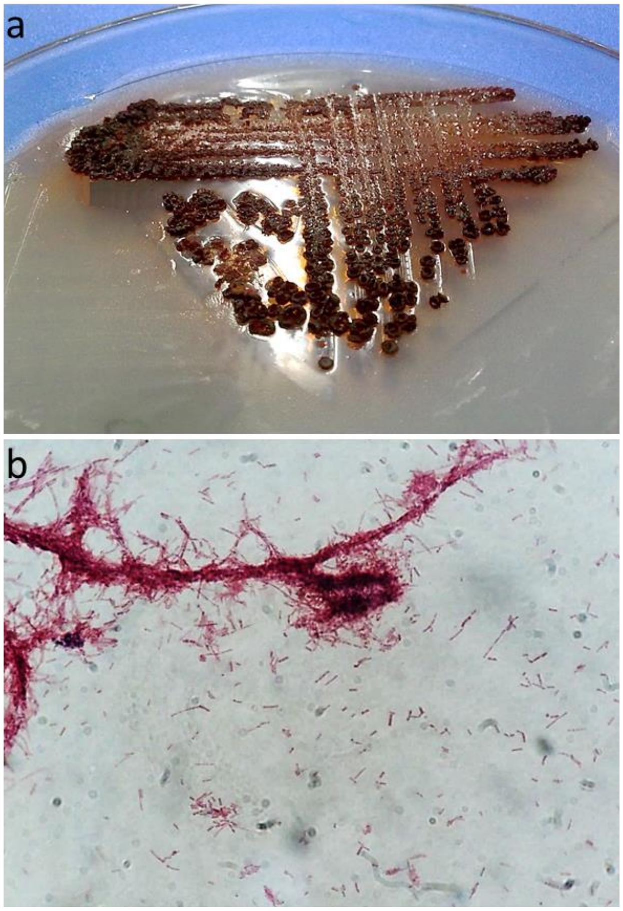

Supplementary Figure S1. Morphological and microscopic characterization of *Micromonospora chokoriensis* CR3 (a) Substrate mycelium (b) Gram-staining of the substrate mycelium under oil-immersion 100X magnification

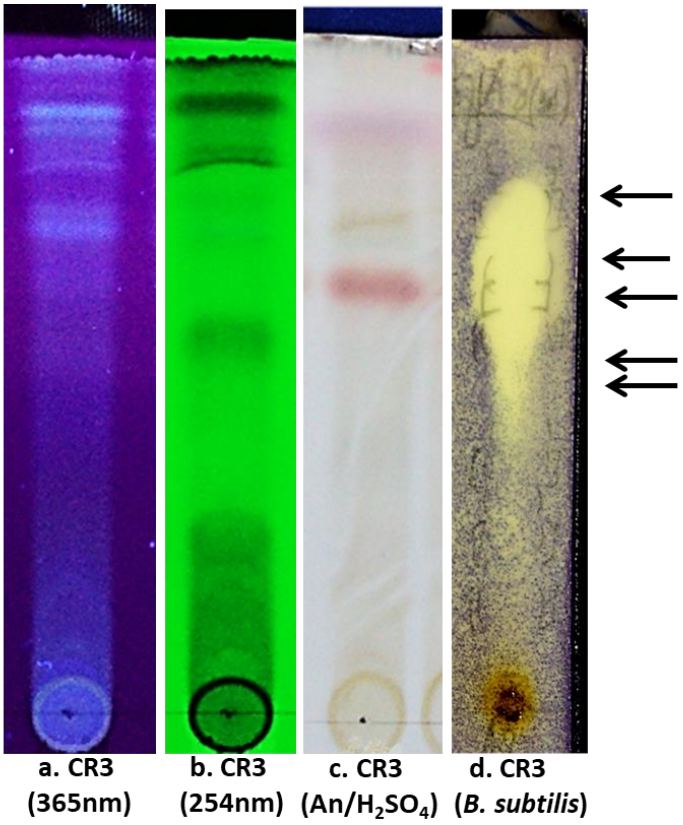

Supplementary Figure S2. Chemical screening of *Micromonospora chokoriensis* CR3 by thin layer chromatography (TLC) and bioautography a) Bands observation under UV 365 nm wavelength b) Bands observation under UV 254 nm wavelength c) Bands observed after staining with anisaldehyde/H_2SO_4 reagent d) Active bands are indicated in bioautography with inhibition (yellow) in places of active bands (shown with arrow). (The picture is closely cropped because other samples not related to this study were also applied on the same TLC plates, which we do not want to disclose as they are related to other unpublished research).
